# D-penicillamine Induced Myasthenia Gravis in Wilson’s Disease: A Case Report

**DOI:** 10.31729/jnma.7607

**Published:** 2022-07-31

**Authors:** Lekhjung Thapa, Monika Thapa, Suman Bhattarai, Abhishek Man Shrestha, Nooma Sharma, Nilshan Rai, Merina Pokharel, Raju Paudel

**Affiliations:** 1 Department of Neurology, National Neuro Center, Maharajgunj, Chakrapath, Kathmandu, Nepal; 2 KIST Medical College and Teaching Hospital, Imadol, Lalitpur, Nepal

**Keywords:** *d-penicillamine*, *myasthenia gravis*, *pyridostigmine*, *Wilson's disease*

## Abstract

Myasthenia gravis is a neuromuscular junction disorder characterised by fluctuating muscle weakness, improved by using anti-cholinesterase drugs. In addition to the autoimmune aetiology, various factors such as infections, surgery, and drugs are known to precipitate the condition. We report a case of a 15-year-old boy with D-penicillamine-induced myasthenia gravis who presented with facial diplegia, dysphagia, and drooling of saliva, 6 years after the initiation of treatment for Wilson's disease. Therefore, clinicians should be more vigilant while prescribing patients with chelating drugs like D-penicillamine with regular monitoring of the new symptoms and keeping a very low threshold for the suspicion of myasthenia gravis.

## INTRODUCTION

Myasthenia gravis (MG) is a disorder of neuromuscular junction classically presenting with fluctuating weakness of muscles of eyes, throat, and extremities, more prominent by the day.^1^ The prevalence of MG in the United States is 20 per 100,000 population which is highest compared to Asia, Europe, and Africa.^1,2^ Serology tests like anti-acetylcholine receptor antibody (AChR-Ab), anti-muscle-specific kinase antibody (Anti-MuSK-Ab).^3^ Electrophysiologic tests like repetitive motor nerve stimulation (RNS), single fibre electromyography (SFEMG); the edrophonium test (sensitivity of 71 % to 95%) supports the diagnosis in the patients with classical symptoms.^4^ This case report describes the case of a patient who developed myasthenia gravis while being treated with D-penicillamine for Wilson's disease.

## CASE REPORT

**Figure 1:**
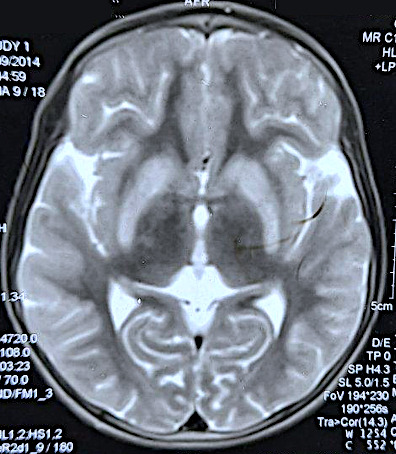
MRI (T2WI) of the brain showing abnormal hyperintensity in bilateral basal ganglia.

A 15-year-old boy from Birgunj, with a known diagnosis of Wilson's disease on D-penicillamine therapy, presented to our Neurology Outpatient Department (OPD) with gradual onset and progressive dysphagia to both solid and liquid food, drooling of saliva, inability to close both the eyes, fluctuating facial weakness with generalised fatigue prominent towards the end of the day, over the course of 2 months. As he had severe dysphagia, he was being fed with a nasogastric tube that was inserted at a local hospital. He had first presented to us 6 years ago with complaints of generalized weakness, spastic limbs, bilateral hands tremor, and drowsiness over a month's duration for which he was evaluated and was diagnosed to have Wilson's disease based on the presence of Kayser-Fleischer (KF) ring, low ceruloplasmin, increased serum copper, increased 24-hour urinary copper, and hyperintensity in abnormal bilateral basal ganglia in T2-weighted (T2WI) magnetic resonance imaging (MRI) (Figure 1).

There were no other significant past medical illnesses or family history. For the past 6 years, he has been treated with D-penicillamine 250 mg twice daily, zinc 20 mg three times a day, and pyridoxine 40 mg once daily.

**Figure 2:**
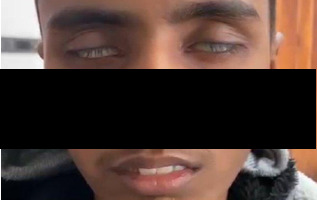
Facial weakness with incomplete closure of mouth and bilateral eyes.

On examination at this visit, the KF ring was absent. He had bilateral facial weakness (Figure 2), generalised hyperreflexia, brisk jaw jerk, and positive cerebellar signs. There was no tongue or muscles atrophy and his neck extensors were normal. Other cranial nerves were normal. His sensory examination was normal. Other systemic examinations were unremarkable.

**Figure 3:**
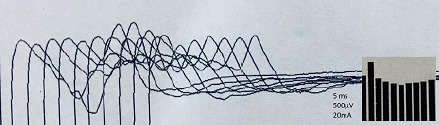
RNS of the left facial nerve (using 10 stimulation trains and 2 Hz frequency) showing >10% decremental response. Stimulation site: Left facial nerve. Recording site: Left orbicularis oculi.

His complete blood count, bood sugar, liver function test, renal function test, thyroid function test, creatine phosphokinase (CPK) and electrolytes (sodium, potassium, calcium, magnesium) were normal. The RNS of the facial nerve (recording from orbicularis oculi muscle) showed a >10% decremental response suggesting postsynaptic neuromuscular junction disorder (Figure 3).

Serum AChR-Ab was positive 14.3 nmol/l (Normal <0.4). Serum anti MuSK antibody report was negative 0.13U/ml (Normal <0.4). Computed Tomography (CT) scan of the chest was done to evaluate for the thymoma was normal.

The diagnosis of drug-induced myasthenia gravis was considered and D-penicillamine was stopped. He was initially treated with pyridostigmine 60 mg twice a day and then later increased to four times a day.

**Figure 4:**
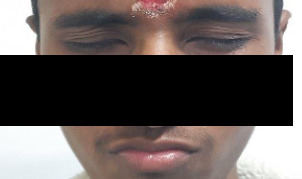
Improvement in facial weakness and complete closure of eyes at 2 months follow-up.

On follow-up at two months, he reported having significant improvement. He could close both his eyes and had substantial improvement in his facial weakness (Figure 4). Despite the mild difficulty swallowing, he could swallow and did not need a nasogastric tube for feeding.

## DISCUSSION

To the best of our knowledge, this is the first case report of D-penicillamine-induced myasthenia gravis in a patient treated for Wilson's disease from Nepal.

D-penicillamine is a pyridoxine antagonist and a potent chelator proven to be very effective in the treatment of the conditions like Wilson's disease, rheumatic arthritis, cystinuria, and lead poisoning.^5^ However, D-penicillamine is known to cause several autoimmune diseases, including MG.^6^ Approximately 1-7% of patients treated with D-penicillamine are reported to develop MG.^6^ The exact mechanism is unknown; however, D-penicillamine has been hypothesized to cause direct modification of major histocompatibility complex (MHC) molecules, as well as peptides on the antigen-presenting cell surface, causing autoimmunity against acetylcholine receptors.^7^

Drug-induced MG predominantly presents with ptosis and diplopia as seen in our patient.^8^ Additionally, our patient had severe dysphagia which was unusual and led us to think that the clinical presentation could be of advanced or inadequately treated Wilson's disease itself. However, KF-ring was absent and the repeated MRI brain was interestingly normal with the resolution of the previous basal ganglia lesion. Therefore, alternative diagnoses of drug-induced MG were considered and evaluated.

MG symptoms are known to usually manifest one month to 8 years after the initiation of D-penicillamine.^8^ Our patient developed myasthenic symptoms after 6 years of treatment for Wilson's disease. Our patient tested positive for AChR-Ab. The test is found to be positive in 80-90% of the generalised MG induced by D-penicillamine.^8^ The antibody titer is uniquely associated with the disease severity, with higher titer manifesting with more severe disease. As our patient had a high AchR-Ab titer, this could explain the severe dysphagia present in our patient. Anti-MuSK-Ab was negative in our case, although it has been reported to be positive in drug-induced myasthenia. However, recent literature emphasises that double seropositivity is rare in drug-induced MG.^9^ RNS study (using 10 stimulation trains and 2 Hz frequency) of the seventh cranial nerve and the response recorded from orbicularis oculi muscle revealed significant decremental response (>10% decrement) in our case. The specificity of RNS has been reported to be very high (about 95%) in both ocular and generalised MG, while the sensitivity is lower (30% in ocular MG and 80% in generalised MG).^10^ Although we could not perform SFEMG and did not perform an Edrophonium test to evaluate the neuromuscular junction (NMJ) disorder in our case, the detailed neurological examination, RNS, and antibody tests we performed are adequate to diagnose MG in routine clinical practice.^11^

Fortunately, withdrawal of the offending drug leads to recovery of myasthenic symptoms. Myasthenic symptoms are reported to subside gradually over 6-10 months after discontinuation of the offending drug in 70% of cases.^6^ It has been assumed that drug-related adverse events that are caused by interference with the NMJ last only for a short period and depend on the half-life of the drug and patient-related factors such as gender, age, renal function, etc. influencing its elimination.^12^ Our patient showed remarkable improvement within 2 months. This rapid improvement is likely due to the pyridostigmine, younger age, and good renal function. We decided to treat him with pyridostigmine because of his severe symptoms and have planned to stop in the next follow-up.

We suggest that clinicians should be more vigilant while prescribing patients with D-penicillamine with continuous monitoring of new symptoms and maintaining a very low threshold for the suspicion of myasthenia gravis. Although discontinuation of offending drugs recovers the symptom, pyridostigmine may help accelerate recovery in such cases.
